# The Electronic Impact of Light-Induced Degradation in CsPbBr_3_ Perovskite Nanocrystals at Gold Interfaces

**DOI:** 10.1021/acs.jpclett.4c00139

**Published:** 2024-03-28

**Authors:** Azmat Ali, Herve Cruguel, Erika Giangrisostomi, Ruslan Ovsyannikov, Mathieu G. Silly, Lenart Dudy, Ute B. Cappel, Emmanuel Lhuillier, Nadine Witkowski, Fredrik O. L. Johansson

**Affiliations:** † Sorbonne Université, CNRS, Institut des Nanosciences de Paris, INSP, F-75005, Paris, France; ‡ Institute Methods and Instrumentation for Synchrotron Radiation Research PS-ISRR, Helmholtz Berlin für Materialien und Energie, Albert-Einstein-Straße 15, 12489, Berlin, Germany; ¶ Synchrotron SOLEIL, l‘Orme des Merisiers, Saint-Aubin, Boîte Postale 48, 9119, Gif-sur-Yvette Cedex, France; § Division of Applied Physical Chemistry, Department of Chemistry, KTH − Royal Institute of Technology, SE-100 44 Stockholm, Sweden

## Abstract

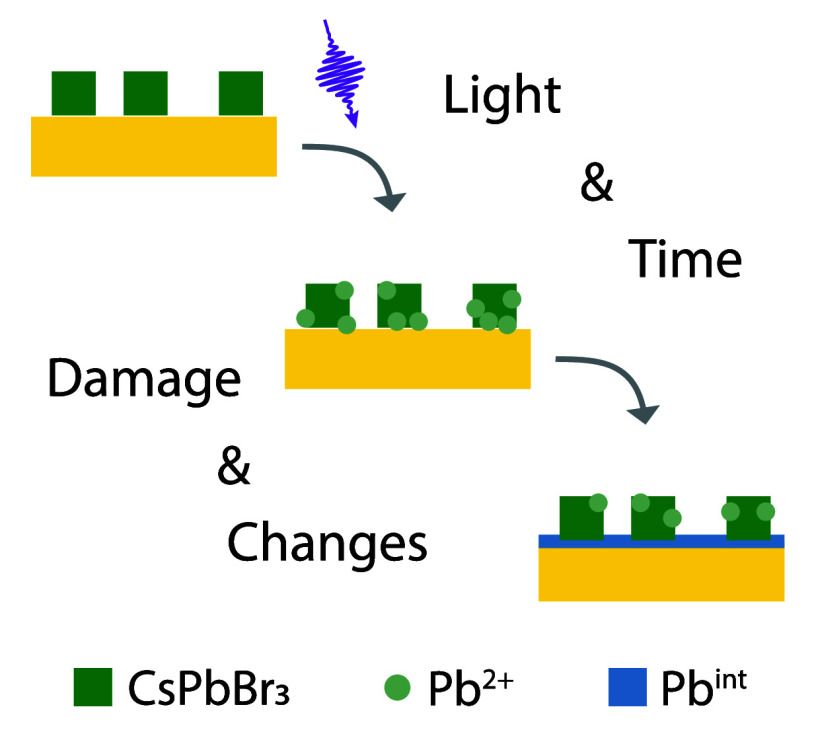

The understanding of the interfacial properties in perovskite devices under irradiation is crucial for their engineering. In this study we show how the electronic structure of the interface between CsPbBr_3_ perovskite nanocrystals (PNCs) and Au is affected by irradiation of X-rays, near-infrared (NIR), and ultraviolet (UV) light. The effects of X-ray and light exposure could be differentiated by employing low-dose X-ray photoelectron spectroscopy (XPS). Apart from the common degradation product of metallic lead (Pb^0^), a new intermediate component (Pb^int^) was identified in the Pb 4f XPS spectra after exposure to high intensity X-rays or UV light. The Pb^int^ component is determined to be monolayer metallic Pb on-top of the Au substrate from underpotential deposition (UPD) of Pb induced from the breaking of the perovskite structure allowing for migration of Pb^2+^.

The exceptional optoelectronic properties of both hybrid and all-inorganic lead halide perovskite materials spurred immense interest for their practical applications in solar cells, scintillators, transistors, light-emitting diodes, lasers, photodetectors and other devices.^1−5^

Recently metal halide perovskites have emerged as a strong contender as new materials for scintillators since they are, compared to traditionally used materials, cheaper and easier to produce, while being efficient and exhibiting good time resolution.^5^ These properties make way for perovskite-based γ- and X-ray detectors, with applications ranging from X-ray imaging, computed tomography for research, medical imaging and security, all the way to space probes.^5−8^ In the mid 1990s CsPbCl_3_ was identified as a promising material for X-ray detection because of its strong luminescence from free excitons under radiation exposure.^9^ In 2013 CsPbBr_3_ was suggested as a potential material for both γ- and X-ray detection,^10^ but problems with purity and material quality have initially hampered the actual realization of a γ-ray detector.

However, development in the synthesis of CsPbBr_3_ not only has made γ-ray detection possible but has shown great energy resolution.^8^

Recently, nanosized perovskites have gained interest since they can be used for position-sensitive detectors that do not require patterning or postprocessing, processes that often affect the properties of bulk materials.^11^ A nanocrystal design also allows for flexible devices and has shown detection limits about 400 times lower than typical commercial X-ray detectors.^12^ Perovskite nanocrystals (PNCs) can also exhibit quantum confinement effects, offering increased light yield and tunable wavelength emission depending on their size.^13^ Both CsPbBr_3_ PNCs^14^ and nanosheets^6^ have been used in perovskite X-ray detectors with promising results.

However, the stability of these materials is essential for device performance. In typical CsPbBr_3_ scintillators, asymmetric electrodes are used to achieve photovoltaic operation, where high- and low-work-function (ϕ) metal contacts are used on the hole and electron extraction sides respectively and in MSM (metal–semiconductor–metal) design the metals are in direct contact with the perovskite. Common high-ϕ metals are Au and Pt, and low-ϕ metals are Ga and Bi.^8^ A thorough understanding of the interaction between the CsPbBr_3_ perovskite and the metal contact under different exposure conditions is needed. For space-oriented applications, the interfacial properties under ultraviolet (UV) and low energy X-ray exposure are essential.

This is similar in perovskite based solar cells where the common cell design uses Spiro-OMeTAD as a electron transport layer in-between the perovskite and a Au contact layer.^15^ Spin-coating of an organic molecule such as Spiro-OMeTAD can exhibit pinholes through the film allowing for ion migration pathways between the perovskite and the Au.

In this study, we investigate the interfacial stability of CsPbBr_3_ PNCs on Au under near-infrared (NIR), UV, and X-ray irradiation, observing the chemical changes through the evolution of the core level photoelectron spectra. We exploit the LowDosePES setup at BESSY II,^16^ where, due to the combination of a low X-ray flux beamline with a high transmission electron spectrometer and a coupled ytterbium-doped fiber laser, the effects of NIR and UV exposure can be isolated from X-ray induced damage.^17^ We show that exposure to both high X-ray flux and UV light leads to irreversible damage of the PNCs, characterized by the appearance of two new chemical species in the Pb 4f spectra, attributed to metallic Pb and underpotential-deposited Pb on the Au surface, and that, on the other hand, a low X-ray dose and high intensity NIR light have limited effects on the PNCs.

The CsPbBr_3_ PNCs were synthesized using a hot-injection method and spin-coated onto Au thin film substrates (details in the Supporting Information). X-ray diffraction showed a crystalline structure (Figure S1) and a crystal size of 13 ± 3 nm (calculations in the Supporting Information). From UV/vis spectroscopy, the band gap was determined as 2.35 ± 0.04 eV (Figure S2), and X-ray photoelectron spectroscopy (XPS) confirmed the Pb, Br and Cs contents (Figure 1 and Figure S3).

**Figure 1:**
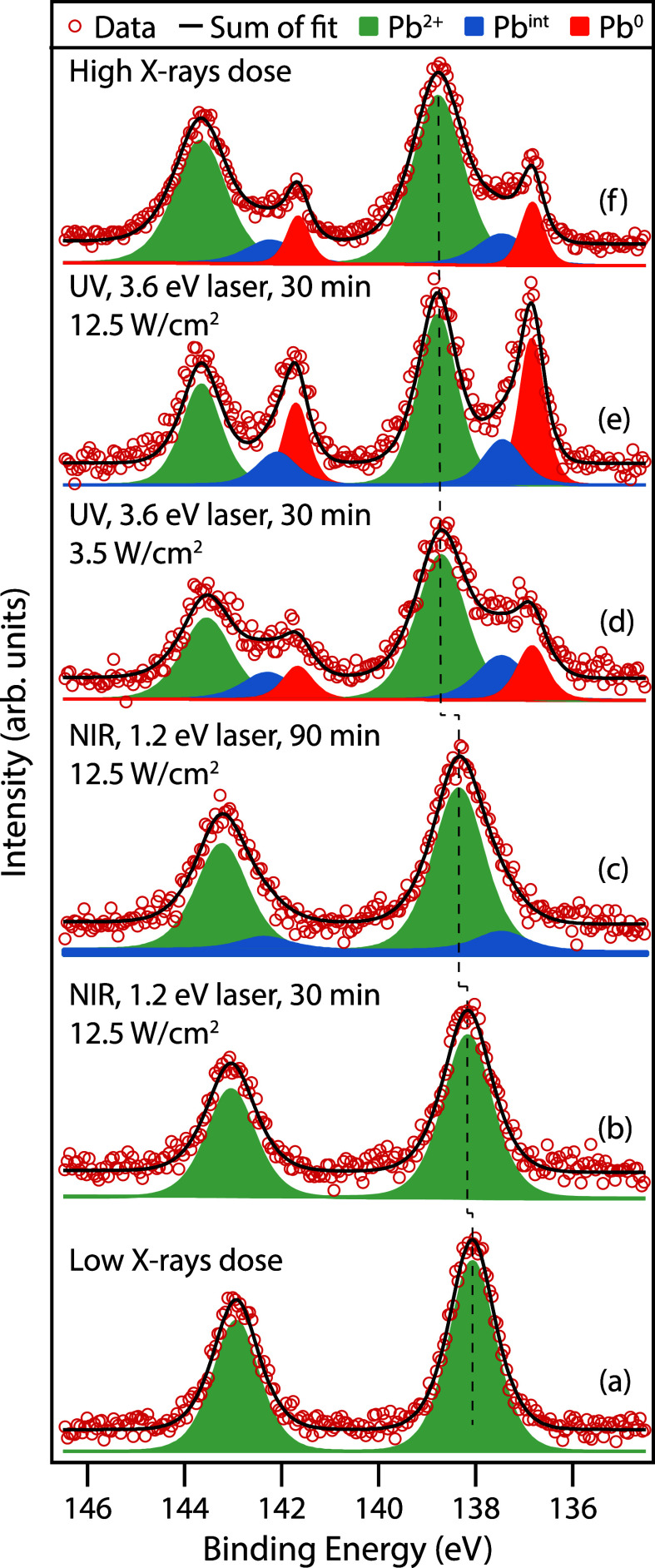
Pb 4f spectra after background subtraction of CsPbBr_3_ PNCs on gold measured with low X-ray flux with a photon energy of 360 eV. (a) fresh spot before light exposure, (b) after 30 min NIR light exposure at 12.5 W/cm^2^ fluence, (c) after 90 min NIR light exposure, after 30 min UV light exposure at (d) 3.5 W/cm^2^ and (e) 12.5 W/cm^2^ fluence, and (f) with high X-ray flux. Each laser exposure is done on a new spot and checked with low X-ray dose XPS prior to exposure.

Figure 1 shows Pb 4f XPS spectra recorded after different irradiation conditions, namely: low and high X-ray flux (10^7^ and 10^10^ photons/s), NIR light from 1.2 eV laser pulses and UV light from 3.6 eV laser pulses at two different fluences (3.5 and 12.5 W/cm^2^). The low X-ray flux measurement was performed at the LowDosePES station at BESSY II using the pseudo single bunch operation with the bending magnet beamline’s mechanical chopper. The high dose X-ray flux measurement is from the undulator beamline TEMPO at the synchrotron SOLEIL using the full multi bunch pattern. NIR and UV light were generated using the MHz laser of the LowDosePES station using its fundamental wavelength (1030 nm, 1.2 eV) for NIR and the third harmonic (344 nm, 3.6 eV) for UV. To achieve similar fluences on the sample for the NIR and UV light exposure, the laser spot size was measured, and a combination of a filter and different laser powers was used. The measurements consist of time series, as indicated in Figures 2 and 3 including two exposure periods, 30 and 60 min, with dark periods before and after. The Pb 4f core-level was continuously measured during the light on periods and the first 15 min of the subsequent dark period, followed by measurements of the other core-level spectra. Figure 1a shows the Pb 4f core-level of the pristine PNCs recorded with a low X-ray flux, which we consider undamaged since the Pb 4f spectrum shows a single spin–orbit doublet assigned to the Pb^2+^ state of CsPbBr_3_.^18^ The corresponding Cs 4d and Br 3d spectra also show a single component assigned to Cs^+^ and Br^–^ of CsPbBr_3_,^18^ shown in Figure S3. The binding energies extracted from least-squares fits of the spectra in Figure 1 can be found in Table 1.

**Figure 2:**
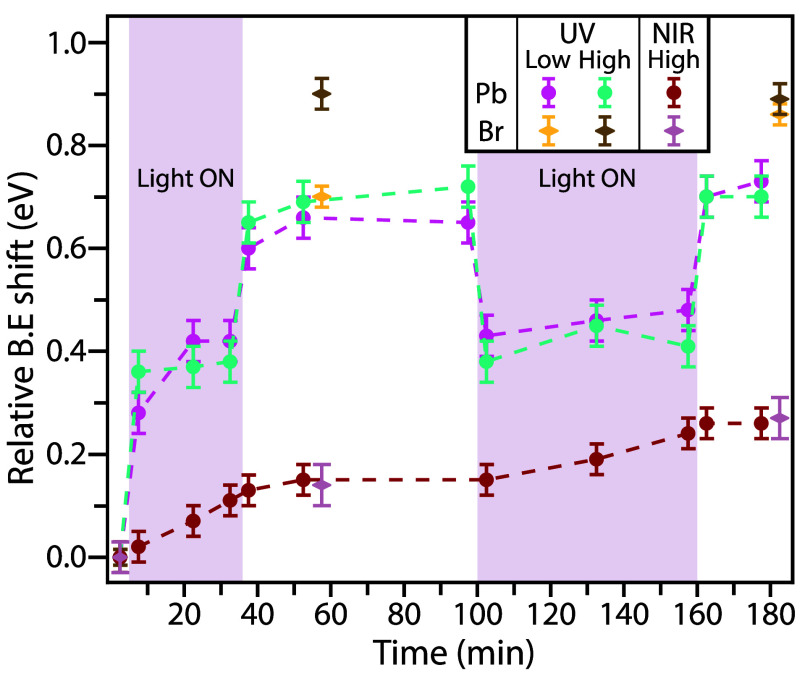
Relative binding energy shifts of the Pb^2+^ component of the Pb 4f spectra and the Br 3d after exposure to NIR light at 12.5 W/cm^2^ fluence and to UV light at 3.5 and 12.5 W/cm^2^ fluences.

**Figure 3:**
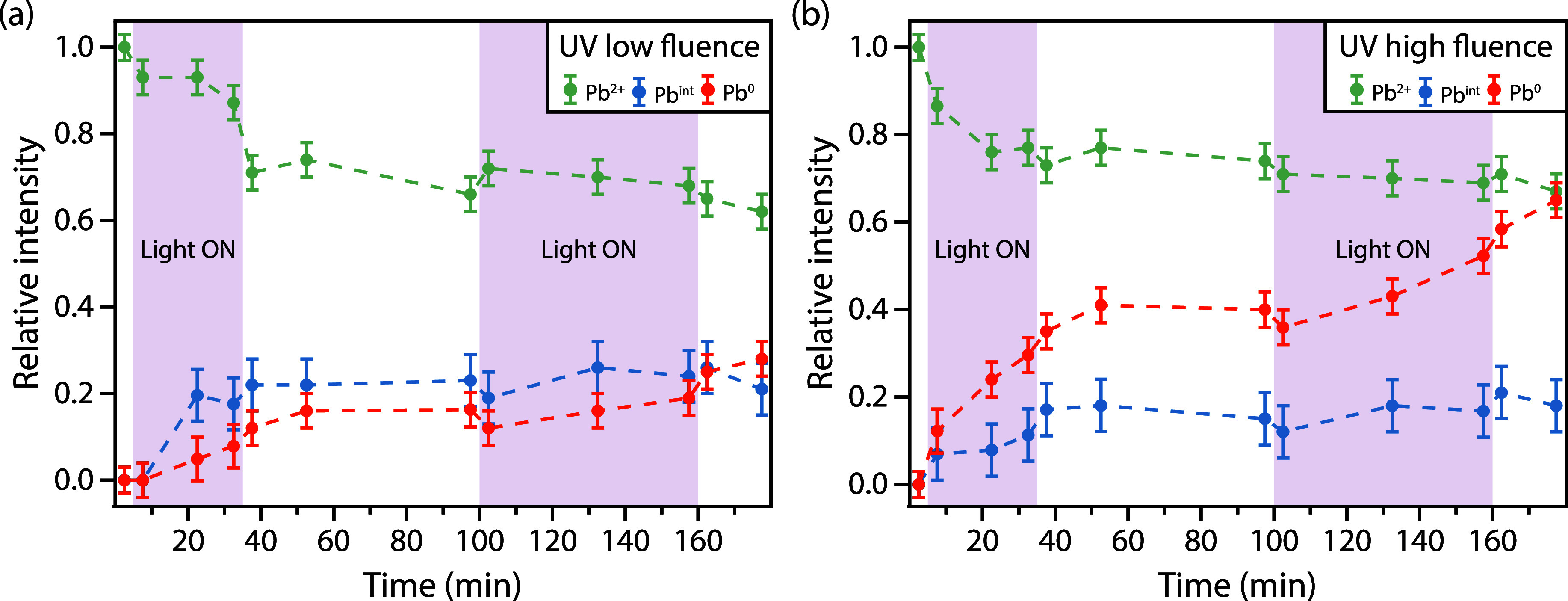
Relative intensities of different components of Pb 4f_7/2_ spectra (intensity of Pb 4f component divided by intensity of the Pb^2+^ component before light exposure) for CsPbBr_3_ PNCs on Au measured with low X-ray flux and photon energy of 360 eV and subject to UV light irradiation with fluence of (a) 3.5 W/cm^2^ and (b) 12.5 W/cm^2^. Shaded areas represent the period when the samples were exposed to the UV light.

**Table 1:** Binding Energies (in eV) of the Three Components Extracted from the Least Squares Fits of the Pb 4f Spectra of CsPbBr_3_ PNCs on Au in Figure 1; Values Are for the 7/2 Spin-Orbit Component

	Pb^2+^ (eV)	Pb^0^ (eV)	Pb^int^ (eV)
Low X-ray flux	138.06	–	–
NIR, 1.2 eV High fluence, 30 min	138.18	–	–
NIR, 1.2 eV High fluence, 90 min	138.29	–	137.45
UV, 3.6 eV Low fluence	138.66	136.84	137.45
UV, 3.6 eV High fluence	138.75	136.84	137.45
High X-ray flux	138.77	136.84	137.45

Figure 1b shows the Pb 4f core-level after 30 min of NIR light exposure (with photon energy smaller than the band gap of the CsPbBr_3_ PNCs) at 12.5 W/cm^2^ fluence and shows only small changes compared to the pristine spectrum. There is only one component which is shifted by 0.12 eV to higher binding energy and no other spectral changes. After 60 min of additional exposure to the NIR light, there is an additional shift in the Pb^2+^ to higher binding energy, and the appearance of a small second component can be observed. The binding energy of this component does not fit with the common degradation product that is metallic lead (Pb^0^),^17,19^ but is somewhere in between Pb^0^ and Pb^2+^, and will be referred to as the intermediate component (Pb^int^) in the further discussion below (the fitting of the Pb^int^ is discussed in the Supporting Information). Figure 1d and e show the Pb 4f core-level after exposure to UV light (with photon energy exceeding the band gap of the CsPbBr_3_ PNCs) for 30 min at the two different fluences of 3.5 W/cm^2^ (low) and 12.5 W/cm^2^ (high). After UV light exposure, the Pb 4f spectrum shows distinct changes: first, the Pb^2+^ has shifted to considerably higher binding energy (Δ = 0.66 and 0.72 eV for low and high fluence, respectively), and second, two new components have appeared. The component at the lowest binding energy is assigned to the formation of metallic lead, Pb^0^.^17,19^ The second new component, between the Pb^2+^ and the Pb^0^, has the same binding energy position as the Pb^int^ observed after sustained NIR exposure and will be discussed in detail below. Figure 1f shows the Pb 4f spectrum recorded using high X-ray flux, approximately 5 orders of magnitude higher than the low X-ray dose, which is noticeably similar to the spectrum obtained after UV light exposure, with Pb^2+^ at higher binding energy, formation of the Pb^0^ and Pb^int^ components. It should be noted that the time taken for the acquisition of the spectrum in Figure 1f is less than one min, meaning that exposure to high X-ray flux affects the PNCs very fast. Therefore, it is clear that PNCs on Au exposed to high X-ray flux for even short times cannot be considered pristine unless careful reference are also recorded.

In Figure 2 the binding energy shifts of Pb^2+^ and Br^–^ for the three different light exposure conditions are presented. Upon NIR exposure, there is a linear shift of the Pb 4f core-level with exposure time, which does not return when the light is turned off. The Br 3d component shows the same shift as the Pb after 30 min exposure, approximately 0.1 eV. In the light off period the binding energy remains constant, and during the second exposure to NIR light the shift continues, again close to a linear trend. After the second exposure the binding energy is steady, and a 0.2 eV shift is measured for both Pb and Br compared to the initial position. Such an NIR-induced change in the binding energy could be from either a chemical shift as a result of damage to the perovskite structure or Fermi level pinning. Since there is a new species formed in the lead by exposure to NIR radiation, as in Figure 1b, a chemical shift cannot be ruled out. Fermi level pinning, on the other hand, is well-known to occur in PNCs.^20^ The amplitude of the shift and the fact that both Pb and Br shift by the same amount make it the probable cause of the binding energy shifts. Even if the origin of the observed shift is not completely elucidated, the present work demonstrates that PNCs are modified by exposure to photons having an energy lower than that of the PNC band gap.

The effect of UV light exposure is distinctly different from that of NIR light exposure. The trend of the Pb 4f binding energy is similar for both fluences, with a large shift during the first exposure period followed by a shift to even higher binding energies when the light is turned off. Similar shifts are seen for the second exposure cycle seemingly on top of a trend of slightly increasing binding energy over time. Looking at Br 3d, the shift is even larger than for Pb 4f, with the high fluence series showing a shift of ∼0.9 eV directly after the first exposure, which is reproduced after the second exposure. For the low fluence series, the shift of Br 3d is different after the two exposures: after the second exposure, it is as high as in the high fluence series, but the shift after the first exposure is lower and equal to the shift of Pb 4f.

From this, we argue that UV light introduces damage to the perovskite directly during exposure which also continues after the exposure stop indicative of a slower process such as ion migration, which is also supported by the formation of Pb^0^ and Pb^int^ (Figure 3). The amount of damage to the perovskite is reflected in the shift of the Br peak, where the full shift occurs immediately for the high fluence exposure and during/after the second exposure for the low fluence.

If we assume that the shift in the Br occurs quickly with UV light exposure, then the shift in the Pb can be seen as resulting from two distinct processes: first, there is a large shift toward higher binding energy (with similar amplitude as Br), followed by a shift to lower binding energies. The latter could be from a surface photovoltage. It is, in fact, a reversible shift, despite the damaged structure, as seen by the reproducibility of the effect during and after the second exposure period. Such surface-photovoltage is compatible with a p-type nature of CsPbBr_3_ PNCs^21^ and with electron trapping on defects at the nanoparticle surfaces under illumination.

The intensity ratio of each of the Pb components (extracted from the least-squares fits) relative to the Pb^2+^ component before UV light exposure is plotted as a function of time in Figure 3. Figure 3a shows the results for the low fluence of 3.5 W/cm^2^, and Figure 3b shows the results for the high fluence of 12.5 W/cm^2^.

As seen in Figure 3a, low fluence UV light exposure leads to a decrease in the relative intensity of the Pb^2+^ component, though this is not initially accompanied by the appearance of additional Pb 4f components. The Pb^int^ component does not appear immediately with UV exposure but after some time, and the Pb^0^ component shows increasing relative intensity during the exposure time. In-between the two exposure cycles, the Pb^2+^ shows an initial decrease and then remains constant, whereas Pb^0^ and Pb^int^ stay constant, within the error margins, to the values at the end of the first exposure. Upon switching the UV light on again, less drastic changes are seen with a seemingly constant Pb^2+^ ratio before and after exposure and an increase in the Pb^0^. After the second exposure, there is again a drop in the intensity of the Pb^2+^ component, though not as large as the first drop, and possibly an increase in the Pb^0^.

With the high fluence UV light, the degradation is stronger, as seen in Figure 3b. Immediately after the UV light is switched on, the Pb^0^ and Pb^int^ components appear, while the intensity of the Pb^2+^ component decreases. There is a subsequent linear increase in the Pb^0^ intensity that persists during the exposure. The Pb^int^ shows a steady increase over the exposure time and the Pb^2+^ a steady decrease. Similarly to the low fluence exposure, the Pb^2+^ has a small drop in intensity when the UV light is turned off; the intensity is then relatively stable with a slow decrease both with and without UV light exposure. The Pb^0^ has a continuous increase even when the illumination is stopped that seems to accelerate with the second exposure. The Pb^int^ is, on the other hand, stable around the intensity reached during the first exposure period.

Comparing low- and high-fluence UV exposure, the behavior is similar for both series with some difference in how fast the intensity ratios change. The Pb^2+^ shows a decrease during the first exposure, then remains relatively stable at approximately 0.6 of the starting intensity. The Pb^int^ exhibits an increase up to a ratio of 0.2, and then relatively stable over time. The main difference comes from Pb^0^, which shows a stronger increase over time under high versus low fluence exposure. Overall, the largest changes are observed during the first 30 min of exposure, suggesting that the pristine structure of CsPbBr_3_ PNCs is more easily damaged than a structure with a higher defect density.

The decomposition mechanism for CsPbBr_3_ under X-ray exposure is described in literature as formation of halide salt (CsBr), halogen gas (Br_2_), and metallic lead (Pb^0^).^22^ In XPS this leads to two components in the Pb spectra: Pb^2+^ and Pb^0^. For PNCs the decomposition can also be foregone by a loss of ligands as discussed in the Supporting Information (Figure S5). A recent study studied the stability of CsPbBr_3_ PNCs of different shapes and sizes under high-energy electron irradiation using transmission electron microscopy, allowing for a direct and detailed observation of Pb^0^ formation. The process involved electron-stimulated desorption of Br and concurrent reduction of Pb^2+^ to Pb^0^, followed by Pb^0^ particles migration to the surface.^23^

The interaction between Au and perovskite thin films has been evaluated in various recent studies. For instance, Cha et al. showed that MAPbI_3–*x*_Cl_*x*_ thin films deposited onto Au exhibit formation of Pb^0^ and attributed it to an interfacial chemical reaction between Au and the perovskite.^24^ Zhao et al. reported on the degradation of MAPbI_3_ thin films with Au deposited on-top by evidencing a shoulder in the Pb 4f spectra, in-between the binding energy of Pb^2+^ and Pb^0^, but without any Pb^0^ contribution. They attributed this to partial charge transfer at the perovskite/Au interface.^25^ Kerner et al. studied the reactions between noble metal contacts with MAPbI_3_ thin films where Au contacts were thermally evaporated on top of the perovskite film.^26^ Using XPS they observed a Pb 4f signal even after evaporation of 50 nm of Au, which they ascribed to diffusion during the deposition process. Continuous XPS measurements after the evaporation showed the Pb 4f core-level shifting to lower binding energies, which they ascribed to underpotential deposition (UPD) of monolayer Pb from Pb^2+^ on the Au surface. UPD is a surface-limited redox process where ions change oxidation state upon adsorption near the equilibrium potential.^26^ This process is known to happen for many ions on noble metal surfaces and is well studied for the Pb^2+^ on Au^27,28^ and is known to occur also for Cs^+^^29^ and Br^–^.^30^

There is a possibility that the Pb^int^ component comes from incorporation of Au in the perovskite structure, in the form of the double perovskite Cs_2_Au_2_Br_6_.^31,32^ Formation of noble metal containing perovskites would start from the interface between Au and the PNCs. Therefore, to determine whether Pb^int^ is a surface contribution we used two different photon energies (360 and 650 eV), where the inelastic mean free path (λ) of the emitted photoelectrons results in information depths (3 × λ) of 2.70 and 4.60 nm respectively,^33^ allowing for differentiating surface from bulk contributions. Figure S4 shows that the Pb^int^ contribution appears for both photon energies and both low and high UV light fluences. However, its relative peak height is smaller in the 650 eV spectra (Table S1), indicating that it originates from the surface. The Pb^0^ component has a stronger photon energy dependence, showing that damage to the PNCs also is a surface effect, with this one scaling with fluence.

Combining this with the aforementioned results, we infer that Pb^int^ is formed only where there is a source of Pb^2+^ close to the Au. In other words, Pb^int^ formation requires activation when the perovskite is pristine. Here, the activation is the exposure of high intensity X-rays or UV light, which damage the perovskite structure, releasing Pb^2+^. An explanation why Zhao^25^ did not see Pb^0^ is that the X-ray anode used for their XPS measurements did not provide a high enough X-ray dose. When the perovskite structure breaks and Pb^2+^ is freed, reduction of Pb^2+^ takes place in UPD of Pb on the Au surface, of which Pb^int^ is the fingerprint. Pb^int^ is formed only in concurrence with Pb^0^ requiring high intensity above gap excitations or ionization to be activated.

As describe above photoinduced degradation of CsPbBr_3_ leads to a loss of Br_2_(*g*), a reaction that is catalyzed through a higher number of trap states,^34^ for PNC this degradation is forgone by loss of ligands leading to a collapse of the surface.^35^ Usually, the ligands are attached to the PNCs via the surface termination of CsPbBr_3_ with either PbBr_2_ or CsBr, with CsBr being more favored.^36^ Under illumination, excitons are generated which subsequently dissociate and diffuse toward the surface of the PNCs where they are captured by surface ligands resulting in their desorption due to their low binding energy.^37,38^ Br vacancies on the nonpassivated ligand-free surface create trap states which promote reduction of Pb^2+^ to Pb^0^.^34^ Comparing the C 1s spectra before and after the UV light exposure (Figure S5), there is a shift of the binding energy comparable to the other core levels and a broadening of the peaks. However, there is no significant change in intensity of the signal, indicating that there is minimal loss of loss of carbon matter meaning that if the ligands are broken, they stay on the surface.

The stable and similar intensity ratio of Pb^int^ after both low and high fluence UV exposure also supports the conclusion that this component stems from the UPD of Pb on the Au surface. Indeed, UPD is self-terminating around monolayer coverage, meaning that once the Au surface is covered with a layer of Pb, the Pb^int^ signal should stay constant. The relative Pb^int^ ratio depends on the nanocrystal coverage in the interaction volume and can thereby be different for different sample preparations. This degradation mechanism is schematically depicted in Figure 4.

**Figure 4:**
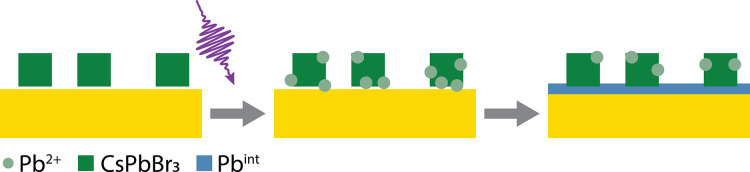
A schematic depiction of the formation of monolayer Pb on Au from UPD after damage to the perovskite structure.

Light-induced thermal degradation, i.e. heating of either the CsPbBr_3_ PNCs or the substrate from the light, could also lead to the formation of Pb^int^ and Pb^0^. If the damage to the perovskite structure arises from heating of the substrate, then it should be stronger closer to the substrate. This is not what is observed in the depth-dependent measurements (Figure S4), which show a larger Pb^0^ formation closer to the surface. The thermal conductivity of perovskites is very low, meaning that locally deposited heat inside the perovskite will not spread out easily and may therefore induce stress in the material.^39,40^ It is probable electron thermalization occurs through Auger cascades, and exciton separation and charge transfer make it unlikely that substantial heating occurs in the CsPbBr_3_ PNCs.

In conclusion, we show that CsPbBr_3_ PNCs on Au, when exposed to UV light or high-intensity X-rays, degrade and form not only the well-known degradation product Pb^0^ but also a second product, Pb^int^, with Pb 4f binding energy between those of Pb^2+^ and Pb^0^. We postulate that this second component comes from the underpotential deposition of Pb on the exposed Au surface. We further show that Pb^int^ does not form when the PNCs are exposed to low-intensity X-rays and that NIR light requires long exposure time at high intensity for Pb^int^ to form. This process can occur for any perovskite and needs to be considered in the design for any situation where the perovskite can degrade and is in contact with Au and potentially other metals.

## Supporting Information Available

Experimental details, sample characterization with XRD, UV–vis and additional XPS spectra including Pb 4f depth profiling. (PDF)
