# Supplementary material for: The Electronic Impact of Light-Induced Degradation in CsPbBr3 Perovskite Nanocrystals at Gold Interfaces
Source: J Phys Chem Lett. 2024 Mar 28;15(14):3721–7. doi: 10.1021/acs.jpclett.4c00139 (PMC11017319; doi:10.1021/acs.jpclett.4c00139)
Supplement: Supplementary file 1 — jz4c00139_si_001.pdf [file jz4c00139_si_001.pdf]

Supporting Information: The Electronic Impact of Light-induced Degradation in $CsPbBr_3$ Perovskite Nanocrystals at Gold Interfaces

Azmat Ali,[†] Herve Cruguel,[†] Erika Giangrisostomi,[‡] Ruslan Ovsyannikov,[‡] Mathieu G. Silly,[¶] Lenart Dudy,[¶] Ute B. Cappel,[§] Emmanuel Lhuillier,[†] Nadine Witkowski,[†] and Fredrik O.L. Johansson [*,§,†,‖]

[†] Sorbonne Université, CNRS, institute des Nanosciences de Paris, INSP, F-75005, Paris, France.

[‡] Institute Methods and Instrumentation for Synchrotron Radiation Research PS-ISRR, Helmholtz Berlin für Materialien und Energie, Albert-Einstein-Straße 15, 12489, Berlin, Germany.

[¶] Synchrotron SOLEIL, l'Orme des Merisiers, Saint-Aubin, Boîte Postale 48, 9119, Gif-sur-Yvette Cedex, France.

[§] Division of Applied Physical Chemistry, Department of Chemistry, KTH – Royal Institute of Technology, SE-100 44 Stockholm, Sweden.

[‖] Present address: Division of X-ray Photon Science, Department of Physics and Astronomy, Uppsala University, Box 516, 751 20 Uppsala, Sweden

[*]fredrik.johansson@physics.uu.se

Experimental methods

Synthesis of $CsPbBr_3$ nanocrystals

Chemicals

1-octadecene (ODE, 90 %, Aldrich), oleylamine (OLA, 80-90 %, Acros), oleic acid (OA, 90 %, Aldrich), cesium carbonate (99.9 %, Aldrich), Lead bromide ($PbBr_2$ > 98 %, Alfa Aesar), hexane (99 %, VWR), ethyl acetate (JT baker), lead acetate (Alfa Aesar, basic).

Cesium oleate

In a three neck 100 mL flask, 0.8 g of Cs_2CO_3 are mixed with 2.5 mL of OA and 30 mL of ODE. The flask is degassed under vacuum for the next 30 minutes at 110 °C. The atmosphere is then switched to Ar and the temperature is raised to 200 °C for 10 min. At this point the cesium salt is fully dissolved. The temperature is cooled down below 110 °C and the flaks is further degassed for 10 min. The obtained gel is used as stock solution.

$CsPbBr_3$ nanocrystals

In a 100 mL three-neck flask, 320 mg of $PbBr_2$ are introduced with 20 mL of ODE. The flask is degassed under vacuum for 60 min at 110 °C. Then, 1 mL of OA is introduced. Once the vacuum level has recovered, 1 mL of OLA is introduced. Quickly after that the lead salt gets fully dissolved. The flask is further degassed for another 30 min at 110 °C. The atmosphere is then switched to nitrogen and the temperature raised to 180 °C. 1.6 mL of cesium oleate solution are quickly added. The reaction is conducted for 30 s before removing the heating mantle and cooling the flask with

ice bath. The content of the flask is centrifuged without addition of non-solvent. The formed pellet is dried and finally re-dispersed in fresh hexane.

Sample preparation

$CsPbBr_3$ perovskite nanocrystals (PNCs) were spin-coated on Au substrate (Au evaporated onto Si wafer) with a speed of 2000 rpm for 20 sec with an acceleration of 100 rpm/sec. The long oleyamine (OLA) and oleic acid (OA) ligands used during synthesis of NCs were exchanged with short and conductive acetate ligands by dipping the NCs thin films in a saturated solution of lead acetate ($(Pb(OAc)_2)$ in ethyl acetate ($Et(OAc)_2$) for 30 sec and then were rinsed in pure ethyl acetate to remove non- reacted precursor, as reported in.[1]

Sample characterization

UV-visible absorption was performed using a JASCO V-730 spectrometer. In-plane X-ray diffraction of spin-coated PNCs of $CsPbBr_3$ on Au was performed using a Smartlab diffractometer using a Cu K-α source.

All XPS measurements except those at high X-ray flux were performed at the Low DosePES station[2] of the PM4 beamline at the synchrotron BESSY II operated by the Helmholtz-Zentrum Berlin, with X-ray flux of $1 \times 10^7 - 1 \times 10^8$ photons/sec. The high X- ray flux (4×10^{13} photons/sec) XPS measurements were performed at the TEMPO beamline at the synchrotron SOLEIL, with an MBS A-1 hemispherical analyzer.[3]

For light exposure experiments, the femtosecond laser system coupled to the LowDosePES station was used. In particular, the fundamental wavelength of the laser was used for IR exposure, and its third harmonic (344 nm) was used for UV exposure. All core levels were measured, before during and after laser exposure, with a photon energy of 360 eV using the 360 l/mm grating and a C_{ff} value of 1.2.

For the NIR and UV exposure, the measurements consist of a time series including two exposure periods, 30 and 60 minutes, with dark periods before and after. The Pb 4f core-level was continuously measured during the light on periods and the first 15 min of the subsequent dark period, followed by measurements of the other core-level spectra.

Data treatment

The binding energy scale for all measured core levels were calibrated to the Au $4f_{7/2}$ set to 84.00 eV. They were fitted using Voigt functions with a background deemed appropriate, either polynomial or Shirley-type, using the CasaXPS software (for Figure S6 the fitting were performed using the SPANCF package in IGOR PRO). For the Pb 4f spectra the binding energy position of Pb^0 and Pb^{int} was kept fixed. The position and width for Pb^0 was found by fitting the high fluence UV-exposure spectra where both the Pb^{2+} and the Pb^0 is well separated. With these components fixed, the Pb^{int} component was subsequently added and fitted. Binding energies are within an error limit of 0.05 eV unless otherwise specified.

Supplementary results

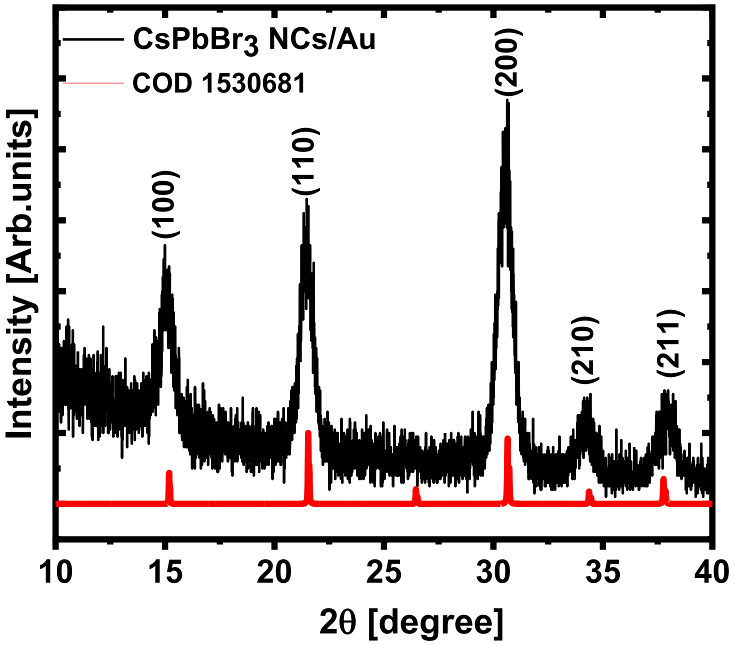

Figure S1. XRD pattern of $CsPbBr_3$ NCs.

The nanocrystal size (*d*) was estimated using the Scherrer equation:

$$d = \frac{K\lambda}{\beta \cdot \cos(\theta)}$$

with the Scherrer constant (*K*) of 0.84, λ of 0.154 nm (Cu Kα radiation), β of 0.60° (fitted from the 200 plane) and θ of 30.53° from the (200) plane position. The average size was estimated by taking all Bragg peak into consideration.

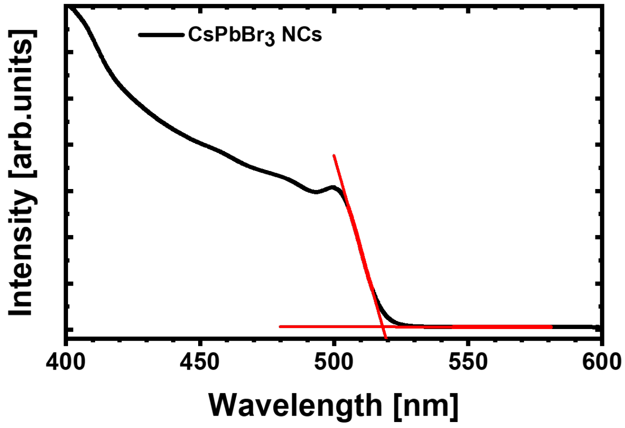

Figure S2. UV-vis spectra of a typical batch of $CsPbBr_3$ NCs from which the band gap calculation was performed, here with the slope at 518 nm. Several batches of nanocrystals were used throughout the experiments and the error estimations if from the slightly varying bandgap from the different batches.

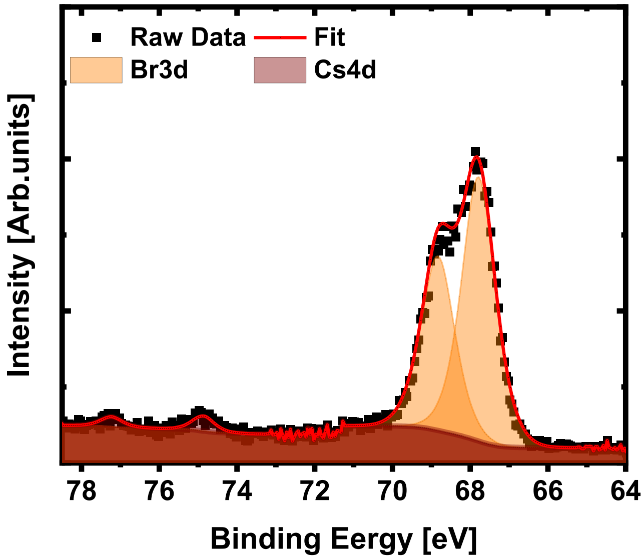

Figure S3. Cs 4d and Br 3d spectra of $CsPbBr_3$ nanocrystals measured with low dose X-rays with a photon energy of 360 eV, fresh spot before laser. With binding energy of 77.25 eV for Cs $4d_{7/2}$ and 68.82 eV for Br $3d_{7/2}$.

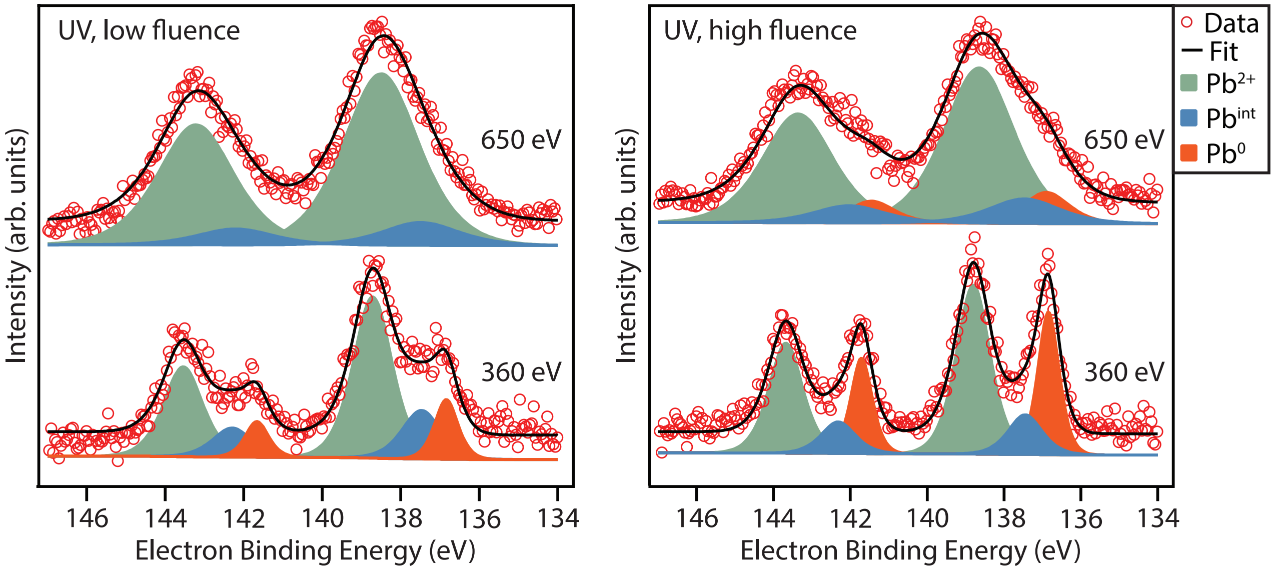

Figure S4. Comparison of Pb 4f spectra of $CsPbBr_3$ nanocrystals measured with 360 and 650 eV photon energy for both low and high UV fluence.

Table S1. Intensity and intensity ratios from the fit in Figure S4. Intensity as area under the peak.

	360 eV			650 eV			360 eV		650 eV	
	Pb^{2+}	Pb^{Int}	Pb^{0}	Pb^{2+}	Pb^{Int}	Pb^{0}	Pb^{Int}/Pb^{2+}	$Pb^{Int}/(Pb^{2+}+Pb^{0})$	Pb^{Int}/Pb^{2+}	$Pb^{Int}/(Pb^{2+}+Pb^{0})$
Low fluence	946	297	205	2109	289	0	0.31	0.26	0.14	0.14
High fluence	992	242	529	2084	335	269	0.24	0.16	0.16	0.14

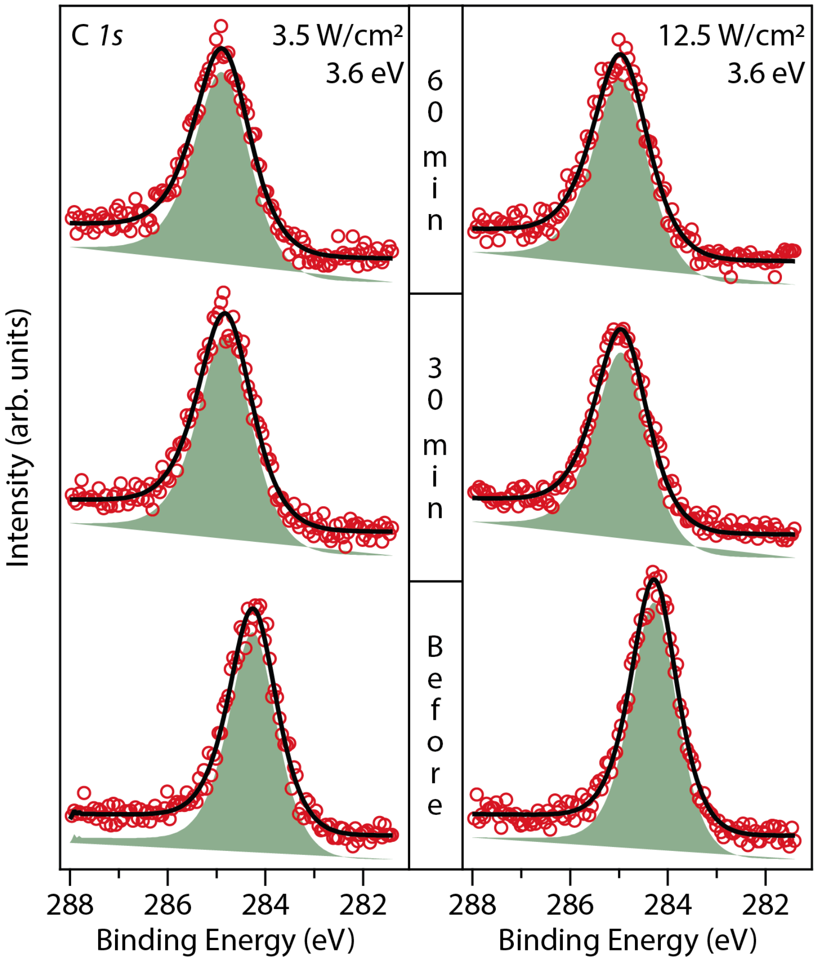

Figure S5. C 1s spectra before and after UV light exposure. Recorded with a photon energy of 360 eV.

To showcase the existence of the intermediate component a comparison of the Pb 4f spectra of the $CsPbBr_3$ nanocrystals on Au and ITO (prepared using the same procedure as for Au) exposed to long and high intensity UV light is presented in Figure S6. The ITO spectrum was fitted using two components, Pb^{2+} and Pb^0, which are well separated. The purple component is Sn 4s from the substrate which shows that the nanocrystal film is not full covering but we also probe the substrate surface where the Pb^{int} should appear. The Au spectra was then fitted using the same parameters as a start, keeping the width for the Pb^0 the same, while allowing the Pb^{2+} to be slightly narrower (1.2 vs 1.4 eV FWHM) to match the peak shape, the energy position was also allowed a free parameter as the two substrates have different surface interactions. As can be seen in the figure, the fit for the ITO is in good agreement with the data whereas for the Au it does not fit well for the region in-between the Pb^{2+} and the Pb^0. looking at the residuals it is clear that there needs to be added an extra contribution in-between the Pb^{2+} and the Pb^0.

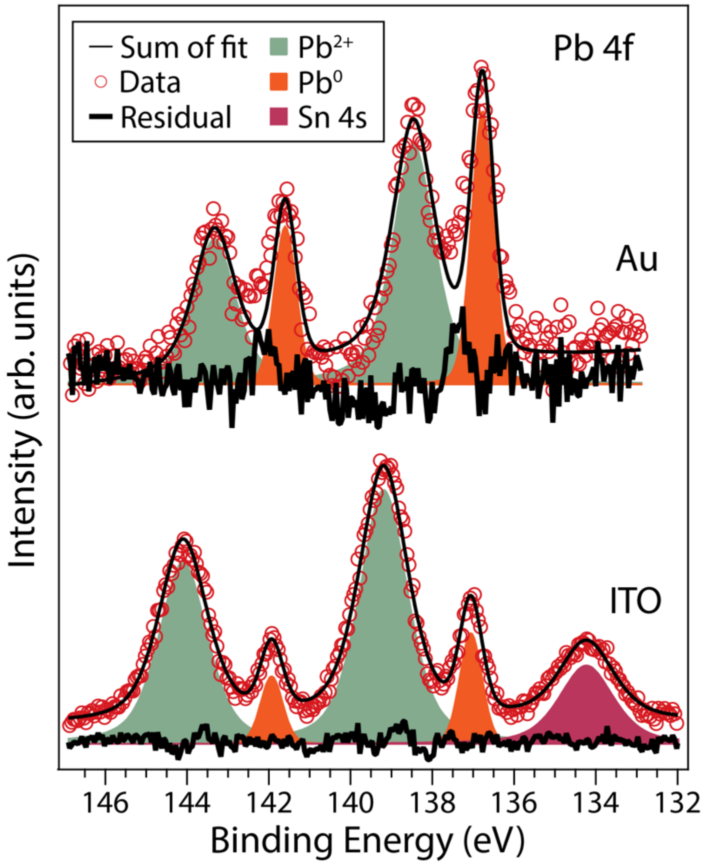

Figure S6. Comparison of Pb 4f spectra of $CsPbBr_3$ nanocrystals on Au and ITO. Recorded using 360 eV photon energy.

References

(1) Amelot, D.; Rastogi, P.; Martinez, B.; Gréboval, C.; Livache, C.; Bresciani, F. A.; Qu, J.; Chu, A.; Goyal, M.; Chee, S.-S., et al. Revealing the Band Structure of FAPI Quantum Dot Film and its Interfaces With Electron and Hole Transport Layer Using Time Resolved Photoemission. *J. Phys. Chem. C* **2020**, *124*, 3873–3880.

(2) Giangrisostomi, E.; Ovsyannikov, R.; Sorgenfrei, F.; Zhang, T.; Lindblad, A.; Sassa, Y.; Cappel, U. B.; Leitner, T.; Mitzner, R.; Svensson, S., et al. Low Dose Photoelectron Spectroscopy at BESSY II: Electronic Structure of Matter in its Native State. *J. Electron Spectros. Relat. Phenomena* **2018**, *224*, 68–78.

(3) Bergeard, N.; Silly, M.; Krizmancic, D.; Chauvet, C.; Guzzo, M.; Ricaud, J.; Izquierdo, M.; Stebel, L.; Pittana, P.; Sergo, R., et al. Time-resolved Photoelectron Spectroscopy Using Synchrotron Radiation Time Structure. *J. Synchrotron Rad.* **2011**, *18*, 245–250.
